# Natural Aristolactams and Aporphine Alkaloids as Inhibitors of CDK1/Cyclin B and DYRK1A

**DOI:** 10.3390/molecules18033018

**Published:** 2013-03-06

**Authors:** Guillaume Marti, Véronique Eparvier, Barbara Morleo, Jessica Le Ven, Cécile Apel, Bernard Bodo, Séverine Amand, Vincent Dumontet, Olivier Lozach, Laurent Meijer, Françoise Guéritte, Marc Litaudon

**Affiliations:** 1 Centre de Recherche de Gif, Institut de Chimie des Substances Naturelles, CNRS, 1 avenue de la Terrasse, 91198 Gif-sur-Yvette Cedex, France; E-Mails: Guillaume.Marti@icsn.cnrs-gif.fr (G.M.); barbara.morleo@icsn.cnrs-gif.fr (B.M.); jessica.leven@gmail.com (J.L.V.); cecile.apel@icsn.cnrs-gif.fr (C.A.); vincent.dumontet@icsn.cnrs-gif.fr (V.D.); francoise.gueritte@icsn.cnrs-gif.fr (F.G.); marc.litaudon@icsn.cnrs-gif.fr (M.L.); 2 Muséum National d’Histoire Naturelle, UMR 7245 CNRS, 63 rue Buffon, Paris 75005, France; E-Mails: bodo@mnhn.fr (B.B.); amand@mnhn.fr (S.A.); 3 Protein Phosphorylation & Human Disease’ group, CNRS, Station Biologique, Place G. Teissier, Roscoff 29680, France; E-Mails: lozach@sb-roscoff.fr (O.L.); meijer@sb-roscoff.fr (L.M.); 4 ManRos Therapeutics, Centre de Perharidy, Roscoff 29680, France

**Keywords:** *Oxandra asbeckii*, *Goniothalamus dumontetii*, *Siparuna* spp., aporphinoids and aristolactams, kinases inhibitors

## Abstract

In an effort to find potent inhibitors of the protein kinases DYRK1A and CDK1/Cyclin B, a systematic *in vitro* evaluation of 2,500 plant extracts from New Caledonia and French Guyana was performed. Some extracts were found to strongly inhibit the activity of these kinases. Four aristolactams and one lignan were purified from the ethyl acetate extracts of *Oxandra asbeckii* and *Goniothalamus dumontetii*, and eleven aporphine alkaloids were isolated from the alkaloid extracts of *Siparuna pachyantha*, *S. decipiens*, *S. guianensis* and *S. poeppigii*. Among these compounds, velutinam, aristolactam AIIIA and medioresinol showed submicromolar IC_50_ values on DYRK1A.

## 1. Introduction

Dual-specificity tyrosine phosphorylation-regulated kinase 1A (DYRK1A) and cyclin-dependent kinases (CDKs) play an important role in the regulation of various cellular processes by phosphorylating serine, threonine and/or tyrosine residues [1]. However, they also can be involved in several human diseases such as cancer and neurodegenerative disorders [1,2,3,4,5]. DYRK1A is a protein kinase with diverse functions, and it is implicated in neuronal development and adult brain physiology. High levels of DYRK1A are associated with neurodegenerative diseases and are also believed to be involved in the neurobiological abnormalities observed in Down-Syndrome, such as mental retardation [5]. The cyclin-dependent kinases (CDKs) are implicated in viral infections, cancer and neurodegenerative pathologies (e.g., Alzheimer’s and Parkinson’s diseases) [6,7,8]. Cyclin-dependent kinases, which are composed of a catalytic subunit (such as CDK1) and a regulatory subunit (such as Cyclin B), play an important role in the regulation of cell cycle progression. For example, the CDK1/Cyclin B complex is known to govern the entry into M-phase [9,10]. For the discussed reasons, these two families of kinases have been extensively used as targets to identify new pharmacological inhibitors of potential therapeutic interest [11]. In this context, and in continuation of our screening program [12] of plant extracts from French Guiana and New Caledonia for the discovery of bioactive natural products, 2,500 extracts (New Caledonian species) were screened against CDK1/Cyclin B, and 720 extracts (French Guiana species) were screened against DYRK1A. The EtOAc extract obtained from *Goniothalamus dumontetii* (R.M.K. Sauders and Munzinger) [13] was selected for its ability to significantly inhibit the activity of CDK1/Cyclin B, while the EtOAc and alkaloid extracts obtained from *Oxandra asbeckii* Pulle (R.E. Fries) and *Siparuna pachyantha* (A.C.Sm.), respectively, were selected for their ability to significantly inhibit the activity of DYRK1A. The selection was then extended to other species of the genus *Siparuna*: *S. decipiens* (A. DC.), *S guianensis* (Aubl.) and *S. poeppigii* (A. DC.). The present paper reports the isolation of 16 compounds, including four aristolactams **1**–**4**, one lignan **5**, and **11** aporphines **6**–**16**, as well as their ability to act as kinase inhibitors.

## 2. Results and Discussion

The chemical investigation of *O. asbeckii* afforded aristolactams AII (**1**) [14] and BII (**2**) [14,15] and velutinam (**3**) (Figure S1) [15,16]. Compounds **1** and **3**, aristolactam AIIIA (**4**) [17] and (−)-medioresinol (**5**) (Figure S2 and S3) [18,19] were isolated from *G. dumontetii*. (+)-Corydine (**6**) [20], (−)-roemerine (**7**) [21,22] and liriodenine (**8**) (Figure S4) [23] were isolated from *S. pachyantha*. (+)-bulbocapnine (**9**) [24,25], (+)-*N*-methyllindcarpine (**10**) [26,27], (+)-actinodaphnine (**11**) [28], liriodenine (8) and (+)-11-methoxynornoelistine (**12**) (Figure S5) [29] were obtained from *S. guianensis*, and the chemical investigation of *S. poeppigii* alkaloid extract yielded lysicamine (**13**) [30], (−)-*O*-methylisopiline (**14**) [31], (+)-*N*-nornuciferine (**15**) [21,32] and liriodenine (**8**). Finally, (+)-boldine (**16**) [33,34] and (+)-*N*-nornuciferine (**15**) were purified from *S. decipiens*. All compounds were identified by comprehensive analysis of spectroscopic and spectrometric data and were compared with data reported in the literature (Figures S1–S5; Table 1 and Table S1) [14,15,16,17,18,19,20,21,22,23,24,25,26,27,28,29,30,31,32,33,34].

**Table 1:** Biological activities of the compounds isolated from the Annonaceae and Monimiaceae plant families.

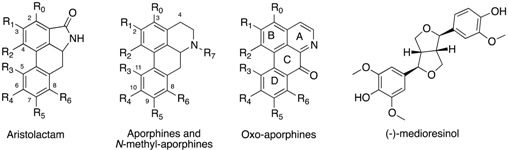										
**Compounds**	R_0_	R_1_	R_2_	R_3_	R_4_	R_5_	R_6_	R_7_	DYRK1A (IC_50_ in μM) ^a^	CDK1/Cyclin B (IC_50_ in μM) ^a^
**Aristolactams**										
aristolactam AII (**1**)	H	OH	OCH_3_	H	H	H	H	H	>30	>30
aristolactam BII (**2**)	H	OCH_3_	OCH_3_	H	H	H	H	H	>30	>30
velutinam (**3**)	H	OCH_3_	OCH_3_	H	H	H	OH	H	**0.6**	**1.5**
aristolactam AIIIA (**4**)	H	OH	OCH_3_	H	OH	H	H	H	**0.08**	**0.2**
***N*-Methylaporphines**										
(+)-corydine (**6**)	H	OCH_3_	OH	OCH_3_	OCH_3_	H	H	CH_3_	>30	>30
(−)-roemerine (**7**)	H	O-CH_2_-O		H	H	H	H	CH_3_	15.0	>30
(+)-bulbocapnine (**9**)	H	O-CH_2_-O		OH	OCH_3_	H	H	CH_3_	>30	>30
(+)-*N*-methyllindcarpine (**10**)	H	OH	OCH_3_	OH	OCH_3_	H	H	CH_3_	>30	>30
(+)-boldine (**16**)	H	OH	OCH_3_	H	OCH_3_	OH	H	CH_3_	>30	>30
**Aporphines**										
(+)-actinodaphnine (**11**)	H	O-CH_2_-O		H	OCH_3_	OH	H	H	>30	>30
(+)-11-methoxynorneolistine (**12**)	H	O-CH_2_-O		OCH_3_	O-CH_2_-O		H	H	**2.5**	>30
(−)-*O*-methylisopiline (**14**)	OCH_3_	OCH_3_	OCH_3_	H	H	H	H	H	>30	>30
(+)-*N*-nornuciferine (**15**)	H	OCH_3_	OCH_3_	H	H	H	H	H	**4.2**	>30
**Oxo-aporphines**										
liriodenine (**8**)	H	O-CH_2_-O		H	H	H	H	-	**3.1**	>30
lysicamine (**13**)	H	OCH_3_	OCH_3_	H	H	H	H	-	**2.4**	>30
**Other**										
(−)-medioresinol (**5**)									**0.1**	**1.3**
6-bromoindirubin-3'-monoxime ^b^									**0.52**	**0.32**

^a^ IC_50_ are mean values from triplicates (the variation is a maximum of 20%); ^b^ positive control; IC_50_ = 3 µg/mL on CDK1-CyclinB for *G. dumontetii* EtOAc extract, and IC_50_ = 3.6 and 1.0 μg/mL on DYRK1A for *O. asbeckii* EtOAc extract and *S. pachyantha* total alkaloid extract, respectively.

Several aporphinoid alkaloids have been previously isolated from *Siparuna* spp. [35,36,37,38], but this is the first time that compounds **6**, **7** and **10**–**16** were described in this genus. In addition, this is only the second time that 11-methoxynornoelistine (**12**) is isolated from Nature [29]. Aristolactams are often found in the species of the genus *Aristolochia* and *Goniothalamus* [39,40], but this is the first time that this type of alkaloid is isolated from an *Oxandra* species [16].

Compounds **1**–**16** were subjected to the CDK1/Cyclin B and DYRK1A kinase inhibition assays (Table 1). Velutinam (**3**), aristolactam AIIIA (**4**) and (−)-medioresinol (**5**) showed the strongest inhibition of CDK1/cyclin B activity, with IC_50_ values of 1.5, 0.2 and 1.3 μM, respectively. The IC_50_ values for inhibition of DYRK1A activity of **3**, **4** and **5** were 0.6, 0.08 and 0.1 μM, respectively. In the aporphine series, (−)-roemerine (**7**), (+)-11-methoxynorneolistine (**12**), (+)-*N*-nornuciferine (**15**), liriodenine (**8**) and lysicamine (**13**) were able to moderately inhibit DYRK1A activity, with IC_50_ values of 15.0, 2.5, 4.2, 3.1 and 2.4 μM, respectively, but these compounds were unable to inhibit CDK1/Cyclin B activity at 30 µM. All other compounds, the aristolactams **1** and **2**, the *N*-methylaporphines **6**, **7**, **9**, **10** and **16**; and the aporphines **11** and **14**, were found inactive at a concentration of 30 µM.

Some very interesting observations can be summarised from these results. In the first series (compounds **1**–**4**), it can be deduced that the presence of a hydroxy group at the C-6 (cf. **4** and **1**) or C-8 (cf. **3** and **2**) positions is critical for achieving inhibition of both CDK1/Cyclin B and DYRK1A activities. In contrast, the presence of a hydroxy group at the C-3 position (cf. **1** and **2**) is not required to inhibit either kinase. Aristolactams, which possess a phenanthrene chromophore, constitute an important alkaloid family due to their potent biological effects [41,42], including anti-inflammatory, anti-platelet, anti-mycobacterial and neuroprotective effects [39]. In particular, aristolactam BII (**2**) exhibited potent cytotoxic activity against human cancer cells [43,44]. In our study, aristolactam AIIIA (**4**) was found to be the most potent compound of the series. This result confirms previous studies in which compound **4** strongly inhibited various kinases such as CDK2, CDK4, and Aurora 2 kinase, with IC_50_ values of 0.14, 1.42 and 2.14 μM, respectively [17,45].

Furthermore, aristolactam AIIIA (**4**) was identified as a new ligand targeting the polo-box domain of Polo-like kinase 1. Bioassays indicated that this natural product could inhibit cancer cell proliferation and induce mitotic arrest at G2/M phase with spindle abnormalities and promote apoptosis [46]. Hedge *et al.* [45] also demonstrated that some lactam derivatives of aristolochic acid were inhibitors of CDK2 activity and that the presence of hydroxy groups at the C-6 and/or C-8 positions results in the enhanced ability to inhibit CDK.

In the second series of compounds (**6**–**15**), only alkaloids **8**, **12**, **13** and **15** were shown to inhibit DYRK1A, but not CDK1/Cyclin B activity, with IC_50_ values in the micromolar range. From these results, it can be deduced that the presence of an *N*-methyl group is detrimental for the interaction with DYRK1A; with the exception of (−)-roemerine, which showed a weak inhibitory activity, all *N*-methylated alkaloids were inactive at 30 µM. Finally, the presence of a methoxy group at the C-3 position (cf. **14** and **15**) abolished DYRK1A inhibition. Liriodenine (**8**), lysicamine (**13**) and *N*-nornuciferine (**15**) are known to possess many biological activities, including anti-microbial, anti-leishmanicidal, and cytotoxic effects on various cancer cells lines [32,47,48]. In addition, Chang *et al.* [49] have shown that liriodenine (**8**) at a concentration of 20 μM induced apoptosis by inhibiting the kinase activity of the CDK1/Cyclin B complex, resulting in G2/M cell cycle arrest. More recently, Chen *et al.* showed that this compound also inhibited the growth of human colon cancer cells and induced G1/S cell cycle arrest [50].

In addition to aristolactams and aporphinoids, the lignan, (−)-medioresinol (**5**), was a strong inhibitor of both kinases. Lignans are known to be cytotoxic and can induce G2/M cell cycle arrest and apoptosis [18,51,52].

## 3. Experimental

### 3.1. General

The NMR spectra were recorded with a Bruker 500 MHz (Avance 500) spectrometer with CDCl_3_ or DMSO-*d_6_* as a solvent. ESIMS were obtained on a Navigator mass Thermoquest. HRESIMS were run on a MALDI-TOF spectrometer (Voyager-De STR; Perspective Biosystems). IR spectra were obtained on a Nicolet FTIR 205 spectrophotometer. The UV spectra were recorded on a Perkin-Elmer Lambda 5 spectrophotometer. Specific rotations were obtained in CHCl_3_ with a JASCO P-1010 polarimeter.

The fractionation was performed in a Harrisson Research^®^ Chromatotron using a rotating disk at 800 rpm. The Kromasil analytic and preparative C_18_ columns (250 × 4.6 mm and 250 × 21.2 mm, 5 μm Thermo^®^, with solvent elution at 1 and 15 mL/min) and the SymmetryShield RP18 preparative column (150 × 19.0 mm, 5 μm Waters^®^, with solvent elution at 17 mL/min) were used for HPLC separation using a Waters autopurification system^®^ equipped with a binary pump (Waters 2525), a UV-vis diode array detector (190–600 nm, Waters 2996) and a Polymer Laboratory PL-ELS 1000 ELS detector. Silica gel 60 (35–70 μm) and analytical and preparative TLC plates (Si gel 60 F 254) were purchased from SDS (Peypin, France). All other chemicals and solvents were of analytical grade and purchased from SDS.

### 3.2. Plant Material

Bark of *O. asbeckii* was collected in 2007 by one of us (V.E.) in the dense forest of French Guiana (under the reference CAY-VE-128). The herbarium specimen was deposited at the IRD Center (Cayenne French Guiana) under the reference CAY-VE-110. Bark of *G. dumontetii* was collected in 2006 by one of us (V.D.) in New Caledonia, under the reference DUM-0558 [13]. Leaves of *S. pachyantha*, *S. decipiens*, *S. guianensis*, and *S. poeppigii*, were collected in 2005 in the dense forest of French Guiana. The herbarium specimens were deposited at the IRD Center (Cayenne French Guiana) under the references CAY: SD-4410, MP-1997, MFP-4517 and MP-1991.

### 3.3. Extraction and Isolation Procedures

Barks of *O. asbeckii* (2.0 Kg) and *G. dumontetii* (650 g) were dried in dry room for a week at 20% humidity. The samples were then crushed. Bark of *O. asbeckii* was extracted by maceration in EtOAc (3 × 2 L) at room temperature to yield 19.3 g. The bark of *G. dumontetii* was extracted using the Dionex^®^ ASE 300 automatic extractor with EtOAc (3 × 100 mL) at 40 °C and 100 bar. The combined extracts were concentrated *in vacuo* at 35 °C to yield 11.7 g.

Leaves of *S. pachyantha* (3.1 Kg), *S. decipiens* (2.1 Kg), *S. guianensis* (6.5 Kg) and *S. poeppigii* (1.5 Kg), were dried in a dry room for a week at 20% humidity. The samples were then crushed. The dried and powdered plants materials were soaked separately in an alkaline solution of 25% NH_4_OH and subjected to ethyl acetate extraction (1.5 L) for 12 h before filtration. The resulting alkaline extracts were then partitioned three times with 2% H_2_SO_4_ (250 mL). The aqueous layers were made alkaline (pH 10) with 25% NH_4_OH and partitioned with CHCl_3_ (1.5 L, at room temperature, 30 min) The chloroformic extracts were then washed three times with distilled water (250 mL) and dried with sodium sulphate. The resulting solutions were evaporated to dryness to yield total alkaloids of *S. pachyantha* (1.6 g), *S. decipiens* (3.3 g), *S. guianensis* (12.5 g) and *S. poeppigii* (3.3 g).

The EtOAc extract of *G. dumontetii* (1.3 g) was subjected to flash chromatography using a gradient of CH_2_Cl_2_/MeOH (100:0 to 50:50) as the eluent and a flux rate of 20 mL/min. Twelve fractions were obtained. Fractions 2 and 3 were shown to inhibit CDK1 with IC_50_ values of 0.24 and 0.70 μg/mL. Fraction 2 (202.1 mg) was purified by preparative HPLC using a Kromasil column with an isocratic mobile phase (MeCN/H_2_O 30:70) to yield compounds **4** (6.8 mg, w/w 0.0094%) and **5** (3.3 mg, w/w 0.0045%). Fraction 3 was purified twice. First, 120 mg was subjected to a Kromasil C-18 column with isocratic MeCN/H_2_O (35:65 + 0.1% formic acid) for 30 min followed by 100% MeCN + 0.1% formic acid for 10 min to yield compound **3** (3.5 mg, w/w 0.0048%). Then, 185 mg was separated on a Symmetry Shield RP18 column with isocratic MeCN/H_2_O (35:65 + 0.1% formic acid) for 25 min followed by 100% MeCN + 0.1% formic acid for 10 min to generate compound **2** (21.2 mg, w/w 0.0293%).

The EtOAc extract of the *O. asbeckii* bark (14.1 g) was subjected to flash chromatography using *n*-heptane/CH_2_Cl_2_ (100:0 to 0:100) followed by elution with CH_2_Cl_2_/MeOH (100:0–50:50) at a flux rate of 20 mL/min. Twenty fractions were obtained. Fractions 16 and 17 were shown to inhibit DYRK1A activity with IC_50_ values of 2.0 and 1.3 μg/mL. Fraction 16 was fractionated by flash chromatography using n-heptane/CH_2_Cl_2_ (100:0 to 0:100) followed by elution with CH_2_Cl_2_/MeOH (100:0–50:50) at a flux rate of 20 mL/min to give 25 fractions. The active fraction obtained was purified by TLC by using CH_2_Cl_2_/MeOH (97:3) as the eluent to give compound **2** (3.0 mg, w/w 0.0002%). Fraction 17 was purified twice. First, 1.2 g was fractionated by flash chromatography using petrol ether/CH_2_Cl_2_ (100:0 to 0:100) followed by CH_2_Cl_2_/MeOH (100:0–50:50). Then, 30.4 mg of the active fraction was separated on a Kromasil C-18 preparative column with isocratic MeCN/H_2_O 35:65 + 0.1% formic acid for 30 min to give compounds **1** (1.7 mg, w/w 0.00012%) and **3** (1.0 mg, w/w 0.00007%).

The total alkaloid extracts of *Siparuna* spp. were fractionated on silica using centrifuge chromatography (Chromatotron^TM^, Harrison Research). A solvent gradient from 100% CHCl_3_ to CHCl_3_/MeOH (90/10) was used as the first fractionation step. Alkaloids were isolated by preparative TLC using diverse mixtures (EtOAc/cyclohexane 50:50, CHCl_3_/MeOH 30:70 and CHCl_3_/MeOH 95:5 + NH_4_). (+)-Corydine (**6**) (3.0 mg, w/w 0.00009%), (−)-roemerine (**7**) (2.5 mg, w/w 0.00008%) and liriodenine (**8**) (5.4 mg, 0.00017%) were isolated from *S. pachyantha*. From *S. guianensis*, (+)-bulbocapnine (**9**) (5.7 mg, w/w 0.00027%), (+)-*N*-methyllindcarpine (**10**) (3.5 mg, w/w 0.00016%), (+)-actinodaphnine (**11**) (15.2 mg, w/w 0.00072%), liriodenine (**8**) (20.8 mg, 0.00098%) and (+)-11-methoxynornoelistine (**13**) (3.2 mg, w/w 0.00015%) were isolated. Lysicamine (**13**) (8.0 mg, w/w 0.00012%), (−)-*O*-methylisopiline (**14**) (22.0 mg, w/w 0.00033%), (+)-*N*-nornuciferine (**15**) (11.3 mg, w/w 0.00017%) and liriodenine (**8**) (1.4 mg, w/w 0.00002%) were purified from *S. poeppigii*, and *N*-nornuciferine (**15**) (11.1 mg, w/w 0.00069%) and (+)-boldine (**16**) (5.6 mg, w/w 0.00035%) were purified from *S. decipiens*. The compounds were identified by spectroscopic analysis and compared to data reported in the literature (see supplementary material) [14,15,16,17,18,19,20,21,22,23,24,25,26,27,28,29,30,31,32,33,34].

### 3.4. Preparation and Assay of Protein Kinases

Kinase activities were assayed in buffer A or C (unless otherwise stated), at 30 °C, at a final ATP concentration of 15 µM. Values were background subtracted, and activities were calculated as pmoles of phosphate incorporated during a 10 min incubation. The activities are usually expressed as % of the maximal activity, that is, in the absence of inhibitors. Controls were performed with appropriate dilutions of DMSO.

DYRK1A (rat, recombinant, expressed in *E. coli* as a GST fusion protein) was purified by affinity chromatography on glutathione-agarose and assayed as described [53].

CDK1/Cyclin B was extracted in homogenisation buffer from M phase starfish (*Marthasterias glacialis*) oocytes and purified by affinity chromatography on p9^CKShs1^-Sepharose beads, from which it was eluted with free p9^CKShs1^ as previously described [53]. The kinase activity was assayed in buffer C, with 1 mg of histone H1/mL, in the presence of 15 µM γ-^33^P-ATP (3000 Ci/mmol; 1 mCi/mL) in a final volume of 30 µL. After a 10 min incubation at 30 °C, 25 µL aliquots of supernatant were spotted onto P81 phosphocellulose paper and treated as described above.

## 4. Conclusions

The search for inhibitors of DYRK1A and CDK1 led us to identify some active plants extracts. Afterward, the chemical investigations of *O. asbeckii* and *G. dumontetii* afforded a series of aristolactams of which the aristolactam AIIIA (**4**) was the strongest inhibitor of both CDK1/Cyclin B and DYRK1A activities. Eleven aporphinoid alkaloids were isolated from various *Siparuna* species, most of them for the first time in this genus. Liriodenine (**8**), 11-methoxynorneolistine (**12**), lysicamine (**13**) and *N-*nornuciferine (**15**) were moderate inhibitors of DYRK1A activity, but at a concentration of 30 µM, they did not inhibit CDK1/Cyclin B activity.

## Supplementary Materials

Supplementary materials can be accessed at: http://www.mdpi.com/1420-3049/18/3/3018/s1.
