# Supplementary material for: Natural Aristolactams and Aporphine Alkaloids as Inhibitors of CDK1/Cyclin B and DYRK1A
Source: Molecules. 2013 Mar 6;18(3):3018–27. doi: 10.3390/molecules18033018 (PMC6269938; doi:10.3390/molecules18033018)
Supplement: Supplementary file 1 [file molecules-18-03018-s001.pdf]

Supplementary Materials

Figure S1. Proton NMR spectra of Compound **3** in DMSO-d_6.

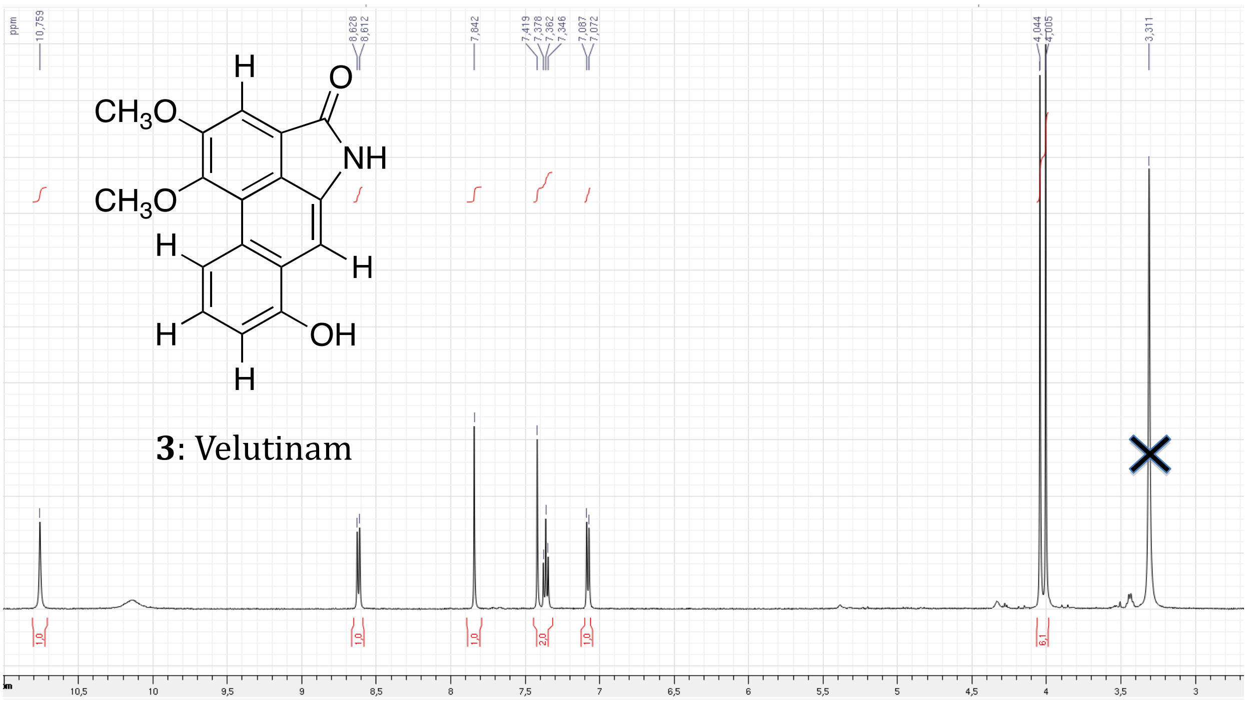

Figure S2. Proton NMR spectra of Compound **4** in DMSO-d_6.

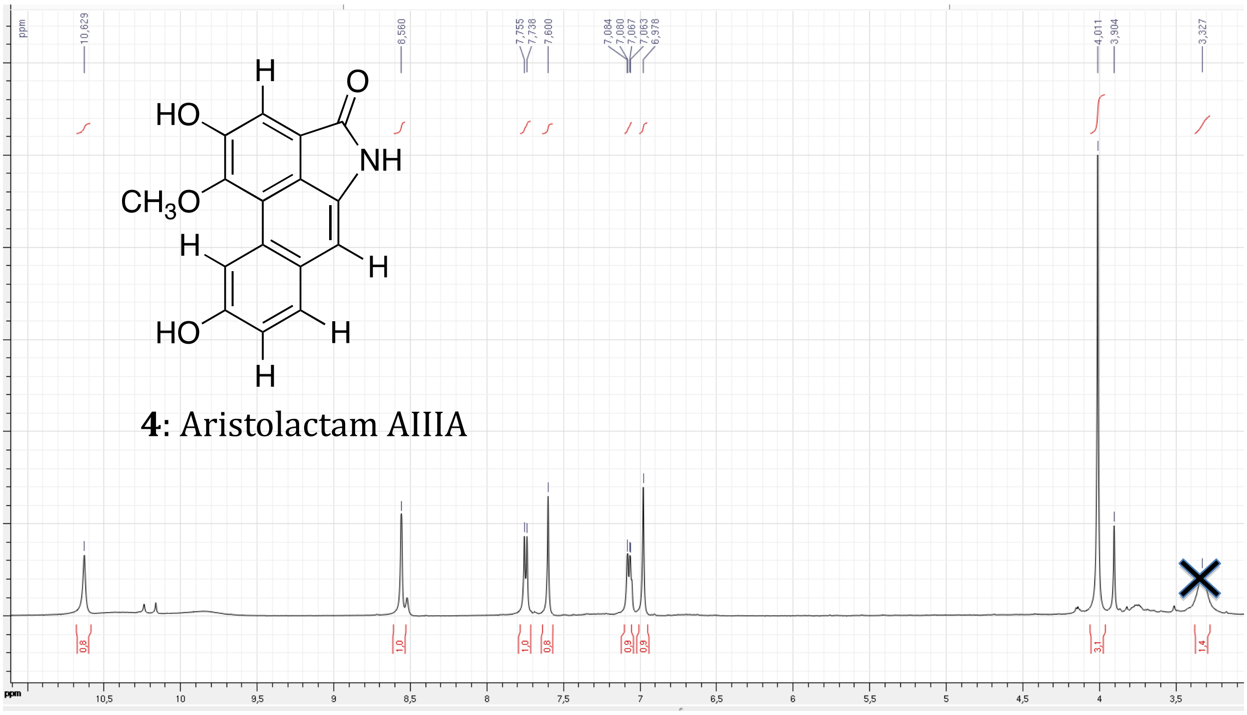

Figure S3. Proton NMR spectra of Compound **5** in $CDCl_3$.

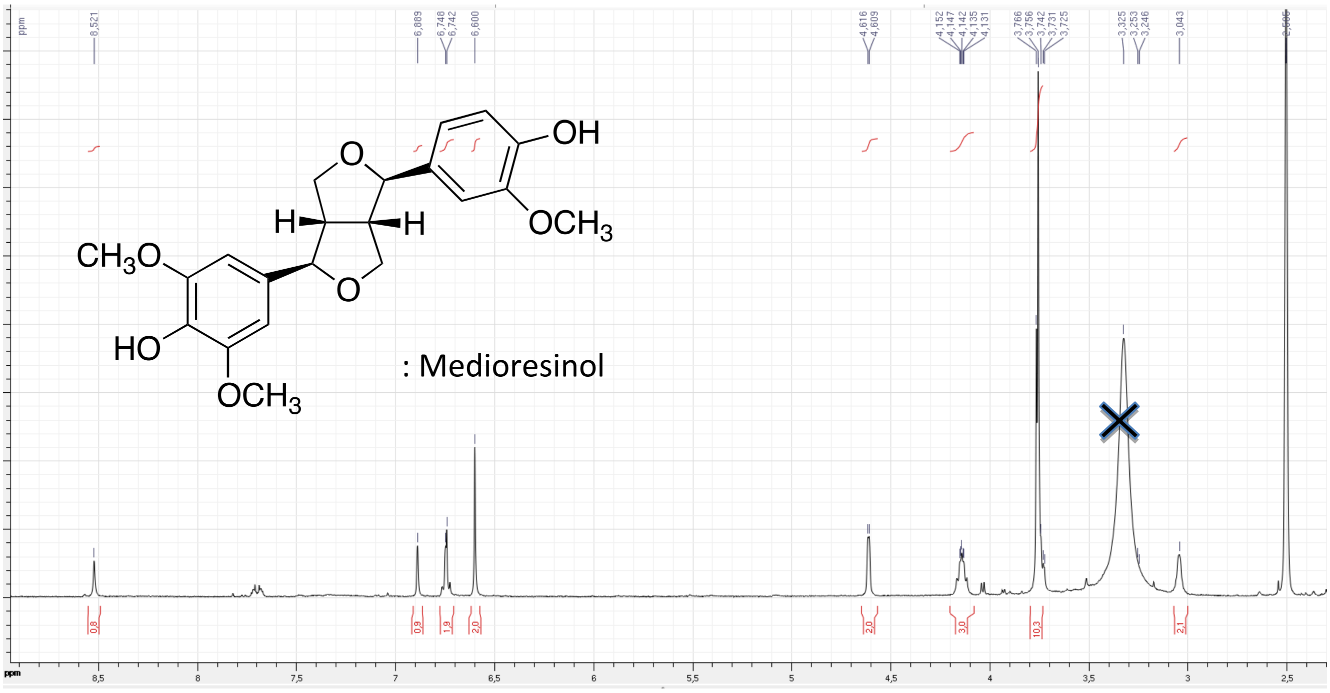

Figure S4. Proton NMR spectra of Compound **8** in $CDCl_3$.

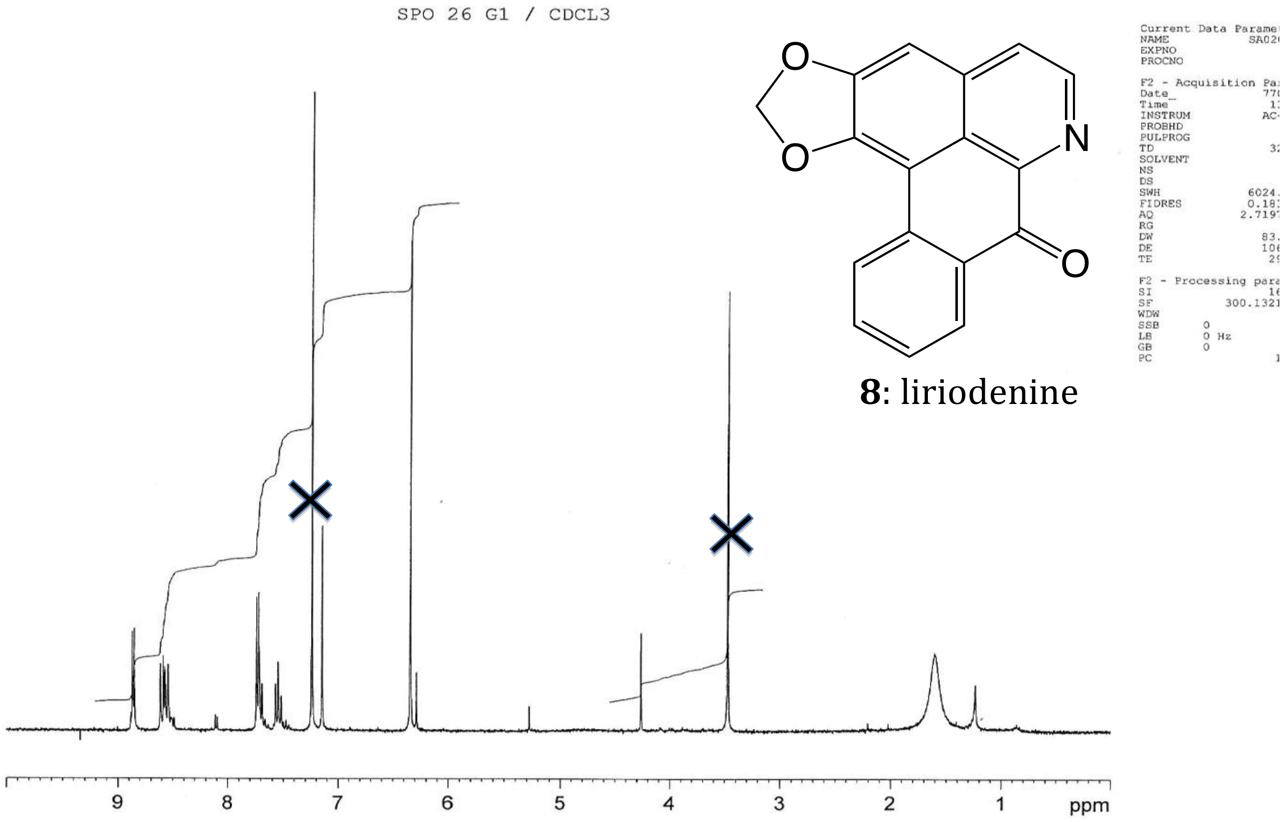

Figure S5. Proton NMR spectra of Compound **12** in $CDCl_3$.

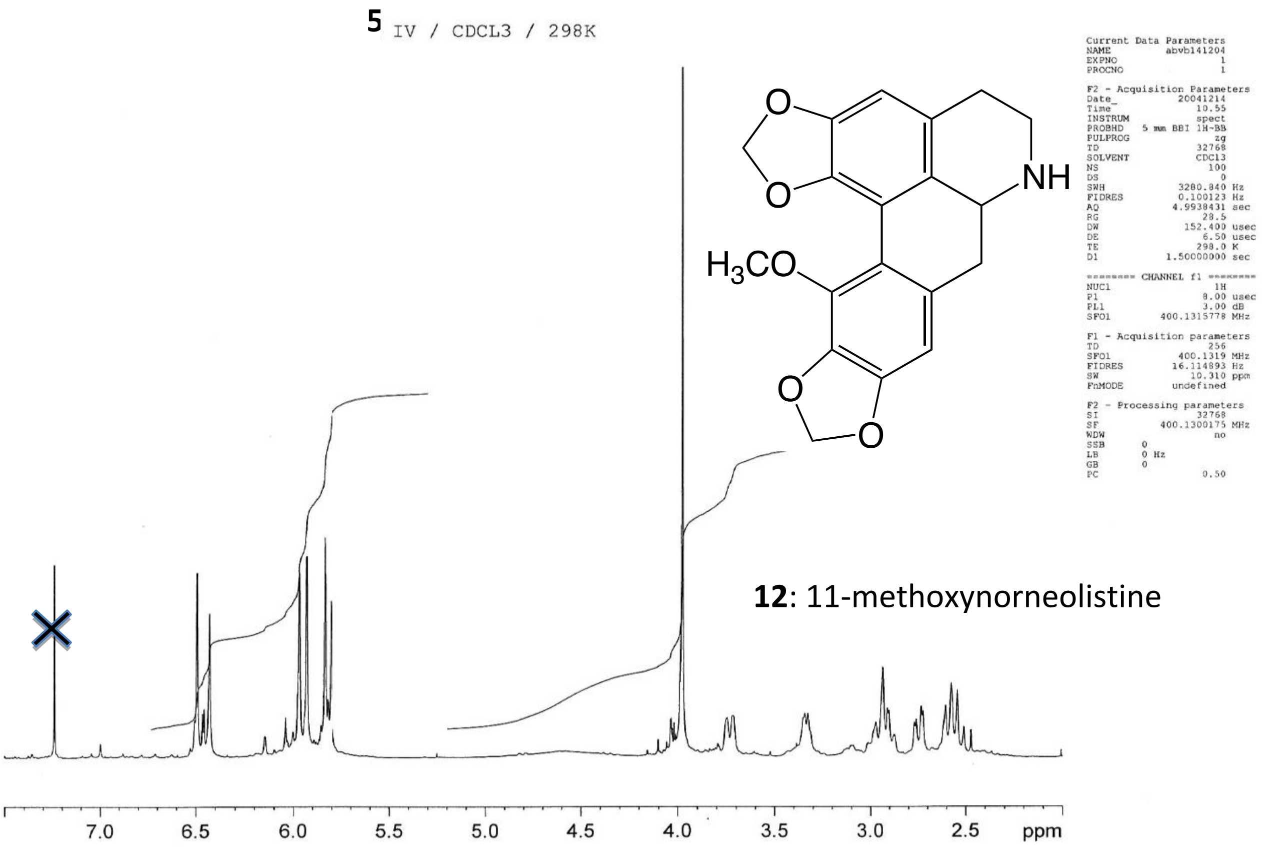

Table S1. Optical rotation of chiral compounds.

Compound	Optical rotation measured	Optical rotation published
(+)-corydine (**6**)	+ 213 (c 0.5 $CHCl_3$)	+ 266 (c 0.36 $CHCl_3$)
(-)-roemerine (**7**)	- 60 (c 0.5 $CHCl_3$)	- 65 ($CHCl_3$)
(+)-bulbocapnine (**9**)	+ 228 (c 0.8 $CHCl_3$)	+ 225 (c 0.85 $CHCl_3$)
(+)-N-methyllincarpine (**10**)	+ 158 (c 0.6 $CHCl_3$)	+ 164 (c 1.0 $CHCl_3$)
(+)-actinodaphnine (**11**)	+ 36 (c 0.9 $CHCl_3$)	+ 32 (c 0.4 ethanol)
(+)-11-methoxynorneolistine (**12**)	+ 48 (c 0.2 $CHCl_3$)	+ 51.2
(-)-O-methylisopiline (**14**)	- 33 (c 0.8 $CHCl_3$)	- in MeOH
(+)-N-nornuciferine (**15**)	+ 35 (c 0.3 $CHCl_3$)	+ 138 (c 0.22 ethanol)
(+)-boldine (**16**)	+ 87 (c 1.0 $CHCl_3$)	+ 108 (c 1.0 ethanol)
(-)-medioresinol (**5**)	- 36 (c 1.0 $CHCl_3$)	- 45.8 (c 0.03 MeOH)
